# In-Situ Study on the Tensile Deformation and Fracture Mechanism of a Bimodal-Structured Mg-Gd-Y Alloy

**DOI:** 10.3390/ma16175978

**Published:** 2023-08-31

**Authors:** Jiangli Ning, Bosong Gao, Jialiao Zhou, Liansheng Chen, Guangze Tang, Shubo Li

**Affiliations:** 1 Key Laboratory of the Ministry of Education for Modern Metallurgy Technology, North China University of Science and Technology, Tangshan 063210, China; gaobosong2022@163.com (B.G.); 13290552663@163.com (J.Z.); kyckfk@ncst.edu.cn (L.C.); 2 College of Metallurgy and Energy, North China University of Science and Technology, Tangshan 063210, China; 3 School of Materials Science and Engineering, Harbin Institute of Technology, Harbin 150001, China; oaktang@hit.edu.cn; 4 Faculty of Materials and Manufacturing, Beijing University of Technology, Beijing 100124, China; lishubo@bjut.edu.cn

**Keywords:** Mg-RE alloy, bimodal structure, in-situ tensile test, slip behavior, fracture mechanism

## Abstract

The as-extruded (EX) Mg-Gd-Y alloy studied here exhibited a bimodal structure, composed of fine dynamic recrystallized (DRXed) grains with random orientations and longitudinal coarse hot-worked grains. The slip analysis showed the DRXed grains exhibited mainly basal slips, while the hot-worked grains exhibited mainly prismatic slips during the tensile deformation. The distribution of geometrically necessary dislocations (GNDs) showed that there was strain partitioning between the fine and coarse grain regions. The hetero-deformation induced (HDI) hardening occurred between the two domains. It improves the strength and strain hardening capability of the alloy, leading to good strength-ductility synergy. Microcracks tended to nucleate at the DRXed grain boundaries, as well as at the interface between the two domains. The calculation of geometric compatibility parameter (*m*’) indicated that strain incompatibility between the adjacent grains induced the crack nucleation. The toughening effect of the fine DRXed grains hindered the crack propagation. However, the major crack formed at the interface between the two domains propagated unstably, due to the high stress concentration and the large crack size, causing the final failure.

## 1. Introduction

Magnesium alloys have the advantages of high specific strength, low density and good recyclability, which have potential for various applications such as automobiles, aerospace and weapons equipment [1,2,3]. For plastic processing or avoiding catastrophic failure during service, high ductility is required for these alloys. However, due to the close-packed hexagonal structure of magnesium alloys, the room-temperature ductility is generally limited, being the obstacle for their applications [4].

The Hall-Petch relationship indicates that grain refinement can significantly improve the strength of alloys [5,6,7]. Ultrafine-grained magnesium alloys could present high strength; however, the ductility usually decreased dramatically [8]. Recently, it has been shown that a good combination of strength and ductility could be achieved by introduction of bimodal structures [9,10,11]. The bimodal grain structure is composed of a large amount of uniform fine grains and a small number of coarse grains, which could improve the strain hardening capability of the alloy. This could lead to excellent mechanical properties for a wide range of applications.

Li et al. [12] conducted compression tests on AZ31 magnesium alloy with uniform and bimodal structures, respectively. The results showed that the ultimate strength, strain hardening rate and fracture strain of the sample with uniform grains were all lower than those of the sample with bimodal structure, though the two samples exhibited close yield strengths. Wang et al. [13] studied the mechanical behaviors of the rolled Mg-3% Al-1% Zn (wt%) alloy before and after annealing. It was found that the formation of bimodal grain structure improved the ductility of the magnesium alloy while maintaining its strength. Li et al. [14] increased the rolling reduction rate from 55% to 85% for Mg-9Al-1Zn alloy, resulting in an optimized bimodal structure. Their study successfully overcame the contradiction that the high reduction rolling process usually improved strength while sacrificing ductility. Peng et al. [15] prepared bimodal structures in Mg-1.0 Gd alloy by adding different contents of Mn elements (0, 0.5, 1.0, 1.5 wt%), achieving improved mechanical properties.

In recent years, it had been found that adding rare earth elements into magnesium alloys could achieve the improvement of strength and ductility by solid solution strengthening, precipitation hardening and texture weakening [16,17,18]. Mg-Gd-Y alloy was one of the most widely studied high-performance rare-earth magnesium alloys (Mg-RE alloys) recently due to the remarkable age-hardening effect [19,20]. Many studies [21,22,23] had been conducted on the relationship between the microstructure and mechanical properties of Mg-Gd-Y alloys processed by different processes, including the study of the effect of bimodal structure [24,25,26].

He et al. [24] reported the mechanical properties of Mg-8Gd-3Y-0.5Zr with bimodal grain size distributions, the morphology of which was claimed to impact the ductility of the alloy. Xu et al. [25] obtained the alloy with bimodal structure by hot extrusion, forced air cooling and artificial aging treatment of Mg-8.2Gd-3.8Y-1Zn-0.4Zr (wt%) alloy. Basal slips and prismatic slips were observed to dominate in the fine recrystallized grains and the coarse hot-worked grains, respectively. The stress transfer between the two domains benefited the toughening of the alloy. Li et al. [26] prepared Mg-8Gd-3Y-0.5Zr (wt%) magnesium alloy with trilayer structure by using extrusion and friction-stir processing. It was pointed out that the back stress caused by deformation incompatibility between the two domains improved the ductility of the alloy.

Generally, in order to improve the ductility of magnesium alloys, it requires a good strain hardening capability to ensure a sufficient uniform deformation stage. Moreover, it is significant to avoid the occurrence of premature failure [27,28,29,30]. However, though the previous researches [24,25,26] considered the impact of strain incompatibility of the heterostructure on the tensile properties, there is still lack of systematically understanding about the influence of detailed slip behaviors on the ductility and toughness of the bimodal-structured Mg-Gd-Y alloys. Therefore, this work studied the evolution and interaction of the slip modes in the different domains, and aimed to disclose their influence on the fracture behaviors, i.e., crack nucleation and propagation of this hetero-structured alloy. For this purpose, an as-extruded Mg-8.24Gd-2.68Y (wt%) alloy without aging treatment was used for the tensile tests at room temperature. Detailed in-situ analysis of slip activities and misorientation analysis based on electron backscatter diffraction (EBSD) were conducted at the grain scale. The potential mechanisms revealed in this work could provide theoretical supplement and guidance for the design of high-performance Mg-RE alloy.

## 2. Experimental Materials and Methods

The material used in the experiment was an EX Mg-Gd-Y alloy. The raw materials were high-purity magnesium and Mg-25Gd (wt%) and Mg-25Y (wt%) intermediate alloys. The materials were melted in a low-carbon steel crucible at approximately 750 °C. CO_2_ + SF_6_ in a ratio of 100:1 served as a protective gas during the melting process. The steel mold was preheated to 200 °C, and then the melt was poured to obtain an ingot with a diameter of 125 mm. The actual chemical composition of the ingot was measured by inductively coupled plasma-atomic emission spectrometer (ICP-AES), which was Mg-8.24Gd-2.68Y (wt%). The ingot was homogenized at 500 °C for 12h and then was subjected to forced air cooling. The homogenized ingot was reheated at 450 °C for 2 h. Then, an extrusion process with a ratio of 16:1 and a pressing speed of 0.3 mm/s was performed to obtain a round rod with a diameter of 32 mm. The extrusion rod was cooled in atmosphere.

The samples for optical observation were cut from the extrusion rod, sanded and polished, and then etched with 4% nitric acid alcohol solution. X-ray Diffraction (XRD) was conducted using a Philips X’Pert diffractometer with CuKα radiation at the voltage of 40 kV and current of 30 mA. The X’Pert Texture software (v4) was used to plot the pole figure (PF) based on the scanning data. The longitudinal section of the extruded specimens was observed and analyzed.

For conventional tensile tests, the tensile specimens were cut into dog-bone shape with 10 mm gauge, 3 mm width and 1 mm thickness. The dimensions of the sample were designed based on the following standard [31]: L_0_ ≥ 5.65 $\sqrt{S_{0}}$ *+* $\frac{B}{2}$, where *S*_0_ is the original cross-sectional area of the gage length. *L*_0_ the gauge length and *B* the sample thickness. The longitudinal direction of the specimens was parallel to the axial direction of the extrusion rod, i.e., the extrusion direction (ED). The samples were then subjected to conventional tensile tests at an initial strain rate of 5 × 10^−4^ s^−1^ using the Instron-3382 testing machine equipped with a video extensometer (AVE 2^®^) at room temperature. The experiments were repeated at least 3 times to test the reproducibility of the results.

For in-situ tensile tests, the tensile specimens were cut into dog-bone shape with 2 mm gauge, 1.5 mm width and 1.5 mm thickness. The longitudinal direction of the samples was parallel to ED. In-situ tensile tests were carried out at room temperature at a constant displacement rate of 1 μm/s using a screw-driven tension stage in the chamber of a scanning electron microscope (SEM, Gemini 300, Shanghai, China). Secondary electron (SE) SEM images and EBSD analysis were taken in the selected area during different strain interrupts during the tensile tests. The acquisition step of EBSD was 0.4 μm, and the analysis software was Channel 5 (v5.0.9.0).

## 3. Results

### 3.1. Initial Microstructure

**Figure 1:**
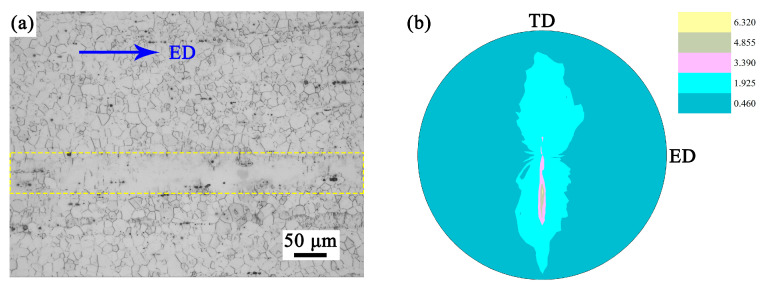
(**a**) The optical micrograph of the longitudinal cross-section, (**b**) the (0001) pole figure of the TD-ED cross-section of the EX sample obtained by XRD.

Figure 1a shows an optical micrograph of the longitudinal cross-section of the EX sample. The horizontal direction in the figure was the ED of the sample. It can be seen that the sample exhibited incomplete dynamic recrystallization after hot extrusion. The coexistence of fine DRXed grains and coarse hot-worked grains formed a bimodal structure. The average grain size of two different types of grains was calculated by an image processing software (Image J v1.53t). The average size of DRXed grains was approximately 14.4 ± 2.4 μm. The region marked by the yellow dotted box was the coarse hot-worked grain. Its average size was about 184 ± 10 μm (represented by the diameter of a circle with equivalent area). The area of coarse grain accounted for approximately 11.5% which was elongated along the ED. As shown in Figure 1b, the EX sample exhibited a weak <$10\overset{-}{1}0$> fiber texture with multiples of random distribution (m.r.d., representing texture intensity) of approximately 5.8.

### 3.2. Tensile Properties

**Figure 2:**
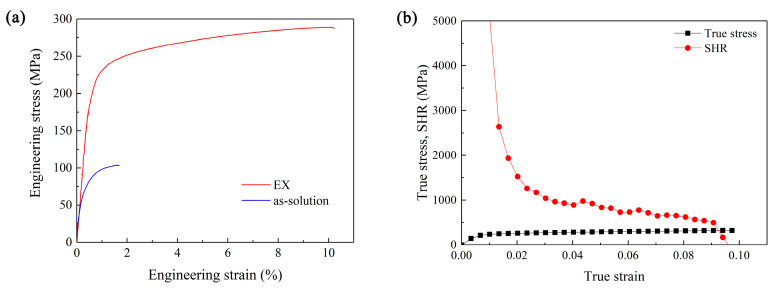
(**a**) The tensile engineering stress-strain curves of the EX and as-solution samples at room temperature, (**b**) the true stress and the SHR curves as the functions of true strain for the EX sample.

Figure 2a shows the tensile engineering stress-strain curve of the EX sample at room temperature. The tensile curve of the solution-treated sample before extrusion (as-solution) is also shown for comparison. The values of tensile properties are shown in Table 1. Figure 2b shows the curve of true stress and strain hardening rate (SHR) of the EX sample as a function of true strain. Formula $d\sigma_{t}/d\varepsilon_{t}$ is used to reflect the SHR of materials. According to the Considère criterion [32], the satisfaction of $\sigma_{t}=d\sigma_{t}/d\varepsilon_{t}$ implies the onset of necking. When the SHR is lower than the true stress, the SHR of the material is insufficient to maintain the uniform deformation of the specimen. From Figure 2b, it can be seen that there was an intersection point between the two curves. This means that the work hardening capacity was almost exhausted after the intersection point, and the material began to undergo plastic instability until fracture.

The SHR curve of the sample decreased rapidly at first and then slowly until it intersected with the true stress curve. This indicates that the work hardening of the material gave full play to the role for maintaining the uniform deformation stage, leading to the decent ductility of the material.

### 3.3. In-Situ Observation of the Deformation and Fracture

**Figure 3:**
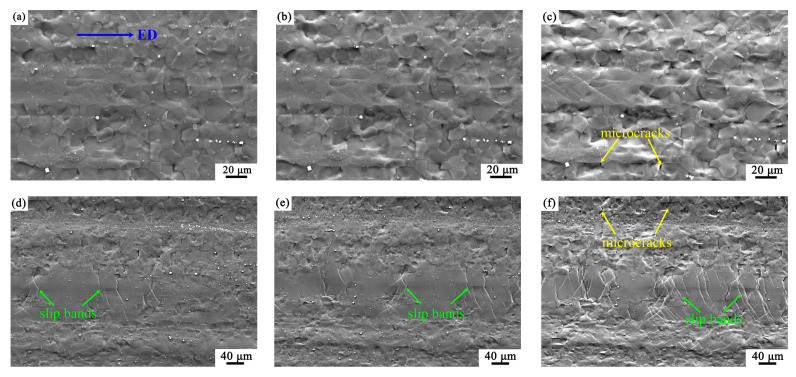
The SEM images of the in-situ tensile specimen show the representative deformation structures at different tensile strains. (**a**–**c**) are the same FG region, corresponding strains are 3.8%, 4.9% and 9.0%, respectively; (**d**–**f**) are the same region containing hot-worked grain, corresponding strains are 3.8%, 4.9% and 9.0%, respectively.

Figure 3 shows the microstructures of the EX sample at different tensile strains. Figure 3a,d correspond to a strain of 3.8%; Figure 3b,e correspond to a strain of 4.9%; Figure 3c,f correspond to a strain of 9.0%. Figure 3a–c show the changes of morphology in the fine-grained (FG) regions during tension. The microstructure in Figure 3a shows a relatively flat morphology. As the strain increased, the morphology of the FG area gradually turned to be roughness, reflecting strain was accommodated between different grains (Figure 3b,c).

Figure 3d–f show the changes of morphology in the coarse-grained (CG) region during tension. The CG area in Figure 3d exhibited a few slip traces. As the strain increased, the slip traces gradually increased to form slip bands (Figure 3e,f). In addition, intergranular microcracks were observed in the FG regions when the strain reached a high value of 9.0%, as shown in Figure 3c,f.

**Figure 4:**
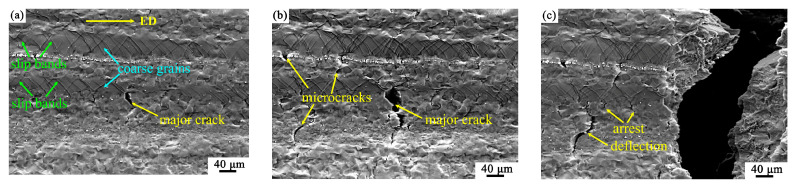
The SEM images of the in-situ tensile specimen show the nucleation and propagation of the cracks at different strains: (**a**) 9.0%, (**b**) 9.4%, and (**c**) 9.8% corresponding to the final fracture of the specimen.

Figure 4 shows the nucleation and propagation of cracks till the fracture of the specimen. When the strain was 9.0%, cross slip bands can be clearly observed in the coarse hot-worked grains, as shown in Figure 4a. A major crack was observed at the interface between the CG and the FG regions. As shown in Figure 4b, as the tension reached the strain of 9.4%, a large number of microcracks can be observed in the FG region and also at the interface between the CG and FG regions. At the same strain, the major crack propagated markedly. When the strain increased to 9.8%, the major crack caused the final fracture of the sample, as shown in Figure 4c. It is noteworthy that the microcracks in the FG region encountered arresting or deflection, showing very limited propagation. The mechanisms of crack nucleation and propagation will be discussed in Section 4.3.

**Figure 5:**
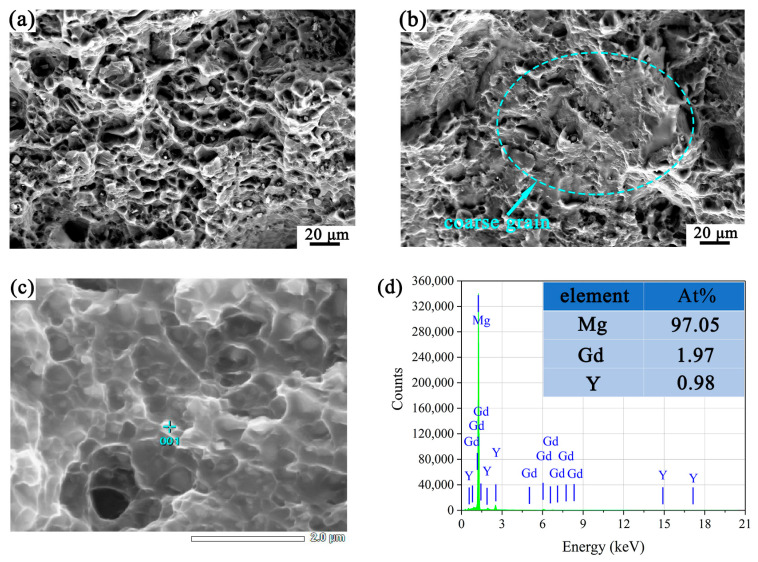
The SEM images show the fracture surface of the tensile specimen: (**a**) the FG region, (**b**) the region containing the coarse hot-worked grain. HAADF-STEM image of (**c**) FG region, and (**d**) the corresponding EDS result.

The tensile fracture surface of the EX sample was shown in Figure 5. A large number of dimples can be observed in the FG region in Figure 5a, indicating that the major fracture mode of the sample was ductile fracture. The presence of second phase particles was observed in some dimples. The composition of the second phase particles was characterized by HAADF-STEM. It can be seen from Figure 5c,d that the second phase particles might correspond to the Mg_5_(GdY) phase [33]. The fracture surface of the CG area was relatively smooth with a few tearing ridges and dimples due to the lack of multisystem slips, as shown in Figure 5b.

### 3.4. Slip Activity during In-Situ Tension

Based on the data of Euler Angles obtained by EBSD, all the theoretical possible slip trace lines were drawn using a matlab program. They were compared with the observed slip traces in the SEM images, and the lines with the coincident directions were determined to be the active slip systems. There may be situations that the directions of multiple slip traces were close to that in the SEM image. In that case, Schmid factor (SF) was taken as the criterion for the selection of the active slip mode, i.e., the one with largest SF value was taken as the active slip system [34,35]. Similarly, since the three slip systems of basal slips were located in the same plane, the one with the highest SF was chosen as the active slip system.

#### 3.4.1. Slip Activity in the Fine-Grained Region

**Figure 6:**
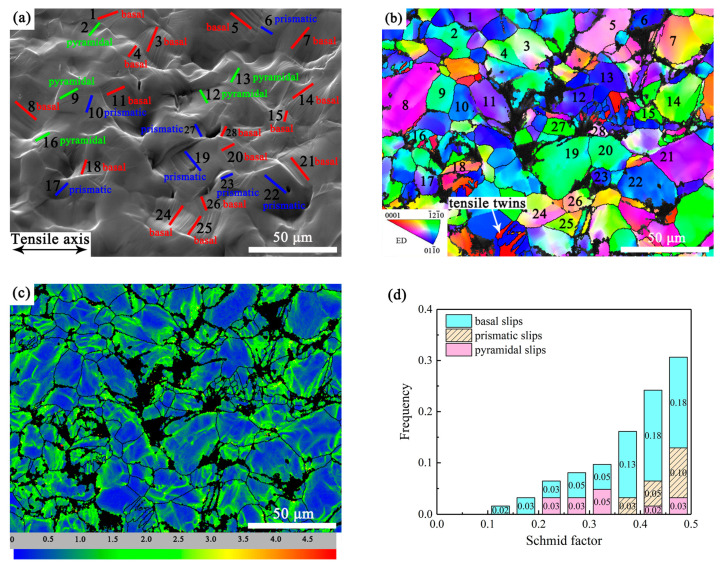
Deformation mode analysis of the FG region at 8.0% tensile strain: (**a**) the SEM micrograph shows the recognized slip traces marked by solid lines and labels. The red solid line represents basal slips, the blue solid line represents prismatic slips, and the green solid line represents pyramidal slips. The numbers 1–28 in (**a**,**b**) correspond to the positions of the grains. (**b**) EBSD orientation map in ED and (**c**) the corresponding KAM map. (**d**) the frequency of the identified slip traces for a particular deformation mode as a function of the SF distribution in the FG region.

The uniform FG region in the EX sample was analyzed at a tensile strain of 8%. It can be observed in Figure 6a that a large number of slip traces appeared in the FG area. This indicates that dislocation slips were the main deformation modes of DRXed grains in the EX sample. The slip modes in the observation area of the FG region included basal slips ({0001}<$\overset{-}{1}\overset{-}{1}20$>), prismatic slips ({10$\overset{-}{1}$0}<11$\overset{-}{2}$0>) and pyramidal slips ({11$\overset{-}{2}$2}<11$\overset{-}{2}$3>). The presence of DRXed grains with different colors in the inverse pole figure (IPF) revealed significant differences in grain orientations, as shown in Figure 6b. Twinning was observed in a few grains (the misorientation of the twin boundary was 86°, indicating tensile twins), but the proportion was low (about 1.84%). The slip modes and the corresponding SF values of 62 slip traces observed in FG regions were statistically analyzed. The results were shown in Figure 6d. The frequency of each slip mode, from high to low, was basal slips (66%), prismatic slips (18%) and pyramidal slips (16%). Most of the slip traces corresponded to high SFs, ranging from 0.25 to 0.5.

The kernel average misorientation (KAM) diagram qualitatively reflects the distribution of plastic strain in grain scale. The larger the KAM value, the higher the degree of plastic strain. The green patches in Figure 6c exhibited concentration of plastic strain, indicating accumulation of dislocations in these areas. It can be seen that the strain distribution in the FG region was relatively uniform, where each grain accommodated a certain extent of strain.

From the Figure 6a,b, it can be seen that a crack nucleated at the junction of grains 19, 27 and 28. Due to the severe deformation near the crack, the confidential index (CI) value in this area was low showing in black in Figure 6b. The relationship between the crack nucleation and the slip activities will be discussed in Section 4.2.

#### 3.4.2. Slip Activity in the Coarse-Grained Region

**Figure 7:**
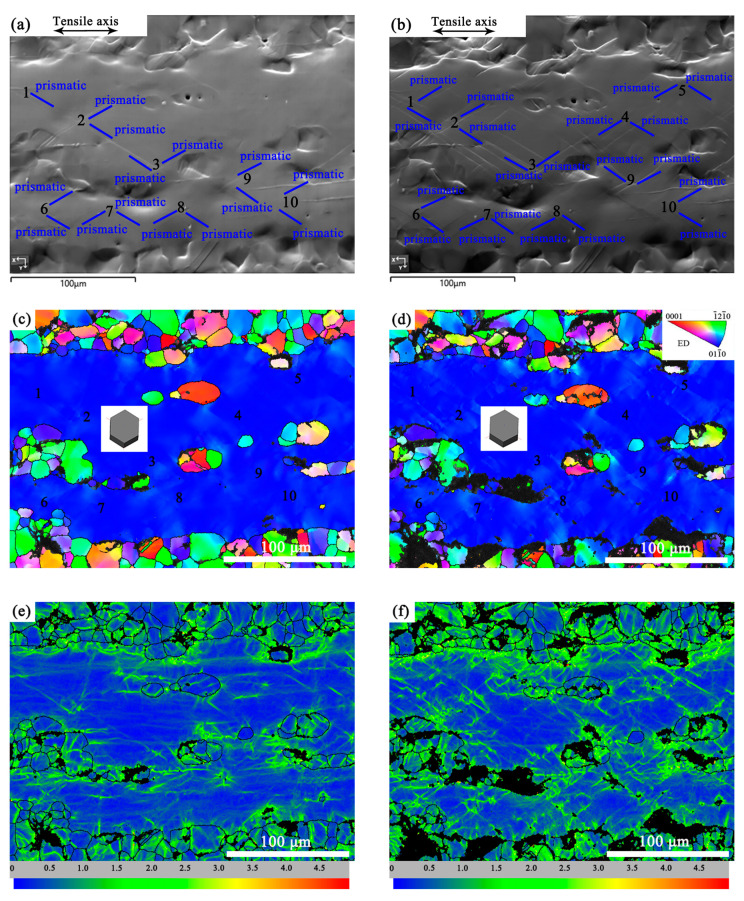
Slip analysis of the CG region at different tensile strains. The SEM micrographs at strains of (**a**) 6.0% and (**b**) 8.0% showing slip traces indicated by solid lines and labels; EBSD orientation maps in ED and the corresponding KAM maps at strains of (**c**,**e**) 6.0% and (**d**,**f**) 8.0%. The numbers 1–10 in (**a**–**d**) correspond to the different positions in the CG region.

A CG region of the EX sample was analyzed at different tensile strains. Figure 7a,b show SEM images of the CG area at the different strains of 6.0% and 8.0%. Figure 7c,d correspond to the IPF maps, while Figure 7e,f correspond to the KAM maps. After the calibration of the slip traces by EBSD data, the deformation mode in the CG area was determined to be prismatic slips ({10$\overset{-}{1}$0}<11$\overset{-}{2}$0>). The slip traces exhibited multisystem prismatic slips. As the strain increased from 6.0% to 8.0%, the number of slip traces increased obviously. The calculated SFs of all activated prismatic slips were greater than 0.38.

The green patches in the KAM images are areas with high local strain. Comparing Figure 7e,f, it can be seen that as the strain increased, the areas of green patches increased. Those correspond to the areas with accumulation of dislocations, which mainly concentrated in the regions adjacent to the interface between the FG and CG domains.

### 3.5. Strain Partitioning in the Bimodal Structure

**Figure 8:**
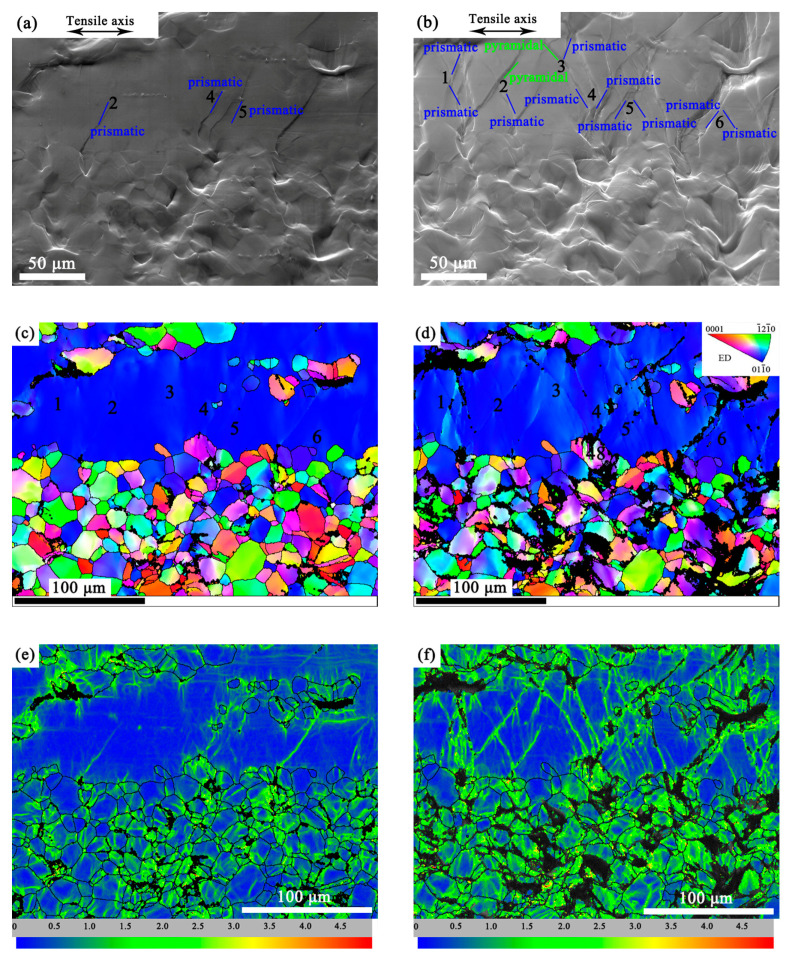
The SEM images at strains of (**a**) 7.0% and (**b**) 9.5%, with solid red lines representing basal slips, solid blue lines representing prismatic slips and solid green lines representing pyramidal slips; EBSD orientation maps in ED at strains of (**c**) 7.0% and (**d**) 9.5%; KAM maps at strains of (**e**) 7.0% and (**f**) 9.5%. The numbers 1–6 in (**a**–**d**) correspond to the different positions in the CG region.

Figure 8 shows the microstructure analysis of the region near the interface between the fine DRXed grains and the coarse hot-worked grains at different strains (7% and 9.5%) during in-situ tension. Figure 8a,b are the SEM images at the strain of 7% and 9.5%, respectively. It can be seen that there were only a few slip traces in the CG area in Figure 8a. These traces were all prismatic slips ({10$\overset{-}{1}$0}<11$\overset{-}{2}$0>) with SFs greater than 0.4. The CG area in Figure 8b exhibited multiple slips, in which most slip traces were identified as prismatic slips ({10$\overset{-}{1}$0}<11$\overset{-}{2}$0>) with SFs greater than 0.39. Moreover, there were a few slip traces identified as pyramidal slips ({11$\overset{-}{2}$2}<11$\overset{-}{2}$3>), with SFs greater than 0.26. Additional slip traces can be observed at positions 1, 3, and 6 in Figure 8b, which did not appear at the same position in Figure 8a. This indicates that as the strain increased, the emerging slip traces increased implying more dislocation slips were activated.

The low CI value in the distortion region was shown as black in the IPF maps, as seen in Figure 8c,d. It can be seen that the area of distortion regions in the FG and CG regions both increased with tensile strain increasing. These distortion zones were caused by high strain concentrations.

**Figure 9:**
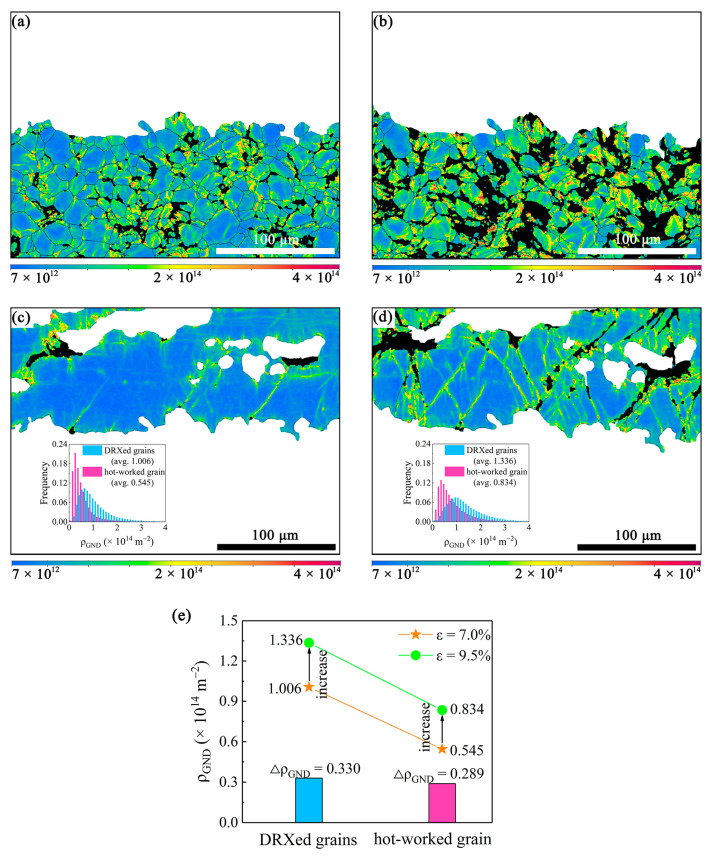
The GND density maps of the DRXed FG region at tensile strains of (**a**) 7.0% and (**b**) 9.5%; the GND density maps of the coarse hot-worked grain at tensile strains of (**c**) 7.0% and (**d**) 9.5%; the inset histograms are the GND density distribution of the two regions at the corresponding strains; (**e**) the increment of GND density (ρ_GND_) of each region as strain increases from 7.0% to 9.5%.

The KAM maps in Figure 8e,f show the change of strain distribution as the strain increased. By comparing the two images, it can be seen that the increase of the area of green patches in the FG region was markedly more than that in the CG region. It can be inferred that as the strain increased from 7.0% to 9.5%, the strain accommodation in the FG region increased more than that in the CG region. Furthermore, a quantitative analysis of the variation of dislocation densities was given in Figure 9.

Figure 9a–d show the density maps of GNDs in the FG and CG regions of the bimodal structure at different strains (7% and 9.5%). The areas in Figure 9a–d with colors towards red indicated high GND densities, i.e., high strain concentrations. As the strain increased, the average GND density of the FG region changed from 1.006 × 10^14^ m^−2^ in Figure 9a to 1.336 × 10^14^ m^−2^ in Figure 9b. The average GND density of the CG region changed from 0.545 × 10^14^ m^−2^ in Figure 9c to 0.834 × 10^14^ m^−2^ in Figure 9d. The increasing amounts of average GND density in the FG and CG regions were 0.330 × 10^14^ m^−2^ and 0.289 × 10^14^ m^−2^, respectively. As shown in Figure 9e, either the average GND density at the strains of 7% and 9.5% or the increasing amount with the increasing strain, the FG region showed larger magnitude than the adjacent CG region. It can be inferred that during the plastic deformation, the DRXed FG region behaved as the soft domain which was easier to deform than the hard domain of the hot-worked CG region. This causes plastic strain partitioning where the soft domain carries much higher plastic strain than hard domain [36].

## 4. Discussion

### 4.1. Hetero-Deformation Induced Hardening

**Figure 10:**
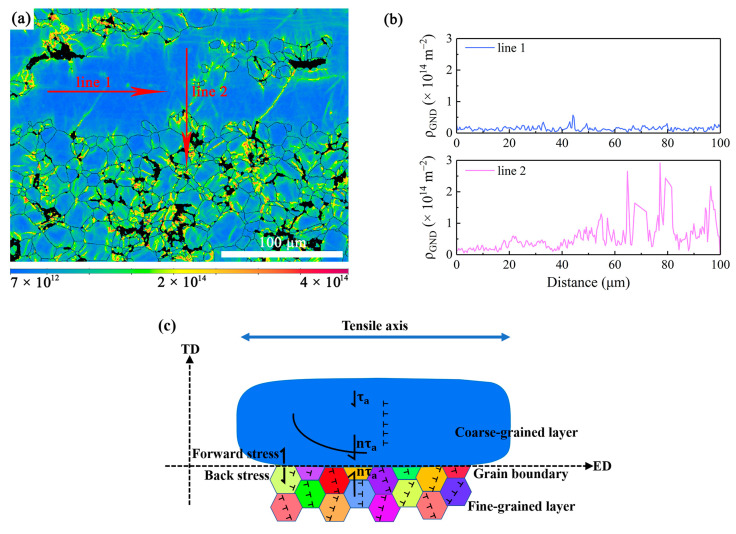
(**a**) The GND map at a strain of 7.0% and the corresponding (**b**) distribution of GND densities along the line 1 and line 2; (**c**) the schematic diagram showing the heterogeneous deformation of the bimodal structure and the HDI hardening.

Figure 10a shows the GND map of the region near the interface between the FG and CG regions at a strain of 7.0%. Draw a horizontal line 1 with a length of 100 μm in the CG area, and draw a straight line 2 with the same length along the direction perpendicular to the interface between the FG and CG regions. Figure 10b reveals the changes in GND density in line 1 and line 2. It can be seen from line 1 that the variation of GND density was relatively uniform in the coarse grain. Only at the position where line 1 intersected with the slip band, a small peak of the GND density emerged. That means the slip bands caused dislocation accumulation. However, as the line 2 went through from the CG to FG regions, there was a significant fluctuation of GND densities at the interface of the two domains, followed by a significant increase in GND densities in the FG region. This indicates that there was a great difference in the magnitude of strain between the FG and CG regions.

In the present bimodal structure, the fine DRXed grains appeared as soft domains, while the coarse hot-worked grains appeared as hard domains, as shown in Figure 9e. This is because the orientations of the fine DRXed grains were close to random distribution (Figure 6b and Figure 8c), which facilitated the initiation of basal slips, as shown in Figure 6d. Since the critical resolved shear stress (CRSS) of basal slip is low, it can be activated under low stress [37,38]. By contrast, the coarse hot-worked grains indicated strong basal-fiber texture, in which only non-basal slips were activated (Figure 7 and Figure 8). The CRSS of either prismatic slip or pyramidal slip is generally higher than that of basal slip [37,38]. Therefore, the coarse hot-worked grains had higher resistance to plastic flow than the fine DRXed grains.

Therefore, stress transfer and strain partitioning could occur between the fine DRXed grains and the coarse hot-worked grains [25,36]. Many studies [36,39,40] demonstrated that GNDs are generated to accommodate the strain incompatibility near the interface between heterogeneous domains. The GND pile-up against the domain boundary would produce back stress in the soft domain and forward stress in the hard domain. This is called HDI hardening [39,40], which collectively produces the strengthening and extra work hardening. The schematic diagram of HDI hardening is shown in Figure 10c. This is an important reason that the present Mg-Gd-Y alloy exhibited a relatively long strain hardening stage and decent ductility. Consequently, the alloy exhibited good combination of strength and ductility.

### 4.2. The Relationship between Crack Nucleation and Dislocation Slip

**Figure 11:**
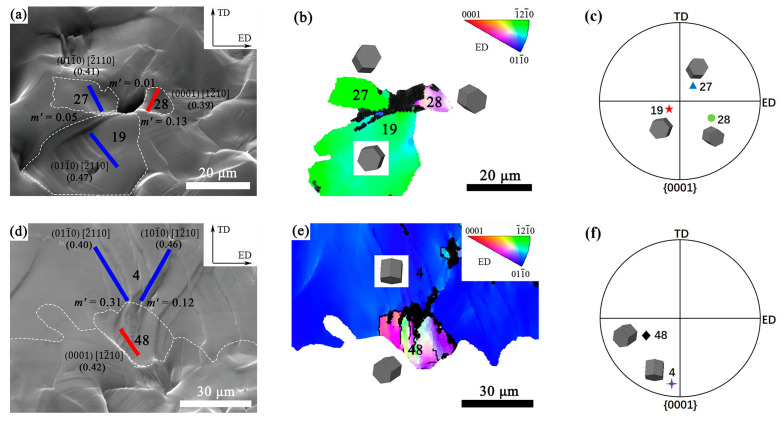
The *m’* analysis of regions prone to crack nucleation: (**a**) SEM image at strain of 8.0%, where solid red and blue lines represent slip traces; (**b**) EBSD orientation map in ED of grains 19, 27 and 28 and (**c**) the corresponding {0001} pole figure at strain of 8.0%; (**d**) SEM image at strain of 9.5%, where solid red and blue lines represent slip traces; (**e**) EBSD orientation map in ED of grains 4, 48 and (**f**) the corresponding {0001} pole figure at strain of 9.5%. The different colored objects in (**c**,**f**) correspond to the orientations of different grains in the {0001} pole figures.

In Figure 11a,b, dislocation slips around the microcrack in the FG region of the EX sample are analyzed. There were crack nucleation at the junction point of grains 19, 27 and 28, extracted from Figure 6. The Euler angles of these three grains were identified to be (123.1°, 166.4°, 24.2°), (137.5°, 17.6°, 10.9°) and (63.7°, 32.0°, 49.7°), respectively. According to the slip trace analysis, grain 19 was identified to perform prismatic slip, in which the activated slip system was (01$\overset{-}{1}$0) [$\overset{-}{2}11$0] and the SF was 0.47. Grain 27 was identified to perform prismatic slip in which the activated slip system was (01$\overset{-}{1}$0) [$\overset{-}{2}11$0] and the SF was 0.41. Grain 28 was identified to perform basal slip, in which the activated slip system was (0001) [1$\overset{-}{2}$10] and the SF was 0.39.

During plastic deformation, dislocations accumulate in grain A and subsequently activate the slip system in adjacent grain B. The geometric relationship of the dislocations within the two adjacent grains can be evaluated using the Luster-Morris factor (*m’*), which is defined in the equation [41]:

*m’* = cos*ψ* cos*κ* (1)

where *ψ* is the angle between the slip direction of the adjacent two grains; *κ* is the angle between the normal slip planes of adjacent grains. Using the parameter *m’*, the geometric compatibility between adjacent grain slip systems may vary from 0 to 1. When *m’* = 1, the slip systems in adjacent grains are completely compatible, and the slip direction and slip plane in each grain are parallel. In contrast, *m’* = 0 indicates that the slip system is completely incompatible, making the slip direction or slip plane orthogonal [42].

Figure 11a shows the *m’* values for possible slip transfer between grains 19, 27 and 28. According to the literature [43], grain pairs with slip transfer generally had high SF values (>0.4) and large *m’* values (>0.7). The calculated data showed that the *m’* values of grain pairs 19–27 (*m’* = 0.05), 19–28 (*m’* = 0.13) and 27–28 (*m’* = 0.01) were very small, implying slip transfer was difficult to occur between them. It can be inferred that incompatible deformation could occur between the adjacent three grains of 19, 27 and 28 during tension. Dislocations were prone to accumulate at these incompatible grain boundaries, resulting in stress concentration and inducing crack nucleation [43,44,45].

From Figure 4, it can be seen that the major crack causing the final fracture of the sample initiated at the interface between the FG and CG regions in the bimodal structure. As seen from Figure 8, there were several points with severe strain concentration at the interface between the FG and CG regions. Those points could be the initiation sites for cracks. Thereby, the slip transfer between the adjacent fine grain and coarse grain adjacent to the interface was studied in Figure 11d–f.

From the bimodal structure in Figure 8d, the grain 48 in the FG region and the position 4 in the CG region were selected. As shown in Figure 11d,e, *m’* values of the slip systems between grain pairs 4–48 were calculated. According to slip trace analysis, the grain 4 was determined to have multiple prismatic slips in which the slip systems were (01$\overset{-}{1}$0) [$\overset{-}{2}11$0] and (10$\overset{-}{1}$0) [1$\overset{-}{2}$10]. The grain 48 was identified to perform basal slip in which the slip system was (0001) [1$\overset{-}{2}$10]. The calculation showed that the *m’* values between grain pairs 4–48 were 0.31 and 0.12, respectively. The small *m’* values implied that the slip transfer between the grain pairs 4–48 was difficult. Therefore, there was poor deformation continuity and compatibility at the local area along the interface between the FG and CG regions. It was a location for stress concentration and could be a potential site for crack initiation [43,44,45].

### 4.3. Effect of Bimodal Structure on Deformation and Fracture

Generally speaking, the tensile deformation of bimodal-structured materials can be divided into three stages [46]. (1) Both the hard and soft domains exhibit elastic deformation similar to that of conventional homogeneous materials. (2) The early occurrence of dislocation slip in the soft domain leads to plastic strain, while the hard domain maintains elastic deformation, resulting in strain incompatibility. Since the deformation at the interface between the two domains is continuous, the soft domain can not deform freely, but is constrained by the adjacent hard domain. Therefore, there will be plastic strain gradient generated in the soft domain near the interface. This strain gradient requires GNDs to coordinate, increasing the strength of the soft domain [47]. The generated synergistic strengthening effect enhances the overall yield strength of the material [48]. (3) Plastic deformation occurs in both the hard and soft domains. However, the soft domain accommodates higher strain compared with the hard domain, causing the occurrence of strain partitioning [49]. The strain gradient will increase with the increase of strain partitioning, resulting in HDI hardening [50]. The HDI hardening helps to delay the necking in tensile deformation, thereby improving ductility [51].

**Figure 12:**
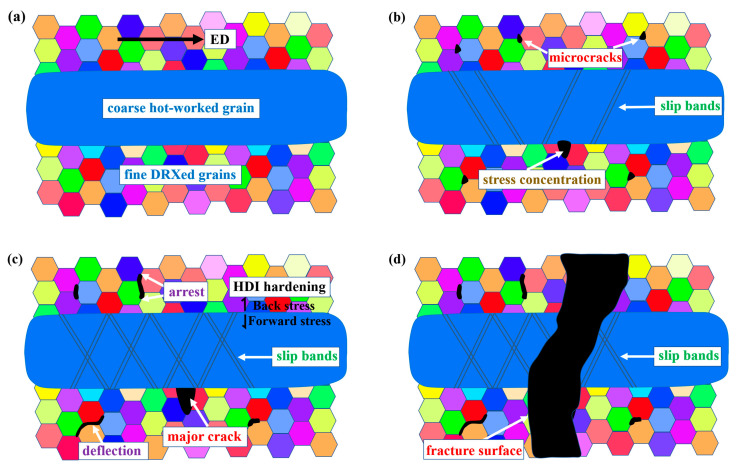
A descriptive model for the tensile deformation and fracture mechanism of the bimodal-structured Mg-Gd-Y alloy showing: (**a**) the microstructure before tension, (**b**) microcracks nucleation, (**c**) propagation of cracks and (**d**) the final failure. The different colors of the hexagonal shapes in the figures represent different orientations of the fine DRXed grains. The large blue ellipse represents the coarse hot-worked grain.

Based on the previous results and analysis, a descriptive model of tensile deformation and fracture mechanism of the EX Mg-Gd-Y alloy with bimodal structure was proposed in Figure 12.

The tensile direction was parallel to ED, as shown in Figure 12a. The small grains with different colors in the figure correspond to the fine DRXed grains, and the blue ellipse in the middle represents the coarse hot-worked grain. In the bimodal structure, the FG region (soft domain) was easier to deform than the CG region (hard domain). In the early stage of deformation, the FG region firstly yielded, while the CG region still maintained elastic deformation. This caused GNDs accumulating and blocking at the interface between the two domains. As the global strain increased, the CG region began to yield.

As the tensile strain increased, crack nucleation occurred between grain boundaries in the FG region and at the interface between the FG and CG regions, as shown in Figure 12b. However, although crack also nucleated in the FG region, the reason for the final fracture of the specimen was the propagation of the crack at the interface between fine and coarse grains.

The reasons why the crack in the FG region did not cause the final fracture of the sample could be attributed to the following points. (1) As shown in Figure 3 and Figure 4, there were many microcracks generated at the grain boundaries in the FG region. These evenly distributed microcracks effectively dispersed the local stress concentration. (2) As shown in Figure 4c, there was deflection when cracks propagated along grain boundaries in the FG region. As the crack surface deflected from the direction perpendicular to the tensor stress, the stress intensity at the crack tip decreased. Finally the cracks stopped inside the FG region. (3) As shown in Figure 9, the fine DRXed grains with random orientations deformed more easily than the coarse hot-worked grains and carried higher plastic strain. The decent ductility of the FG region released the stress concentration of the microcracks effectively and delayed the crack propagation.

According to Figure 11d, strain incompatibility existed near the interface between the fine and coarse grains, as a potential site for crack initiation [43,44,45]. Meanwhile, according to the GND analysis in Figure 9, there was a remarkable difference in the resistance to plastic flow between the FG and CG regions in the bimodal structure. It implies that the domain boundary will be very effective in blocking GNDs. Thus, the GND pile-ups near the interface will cause significant stress concentration, leading to the initiation and propagation of cracks at the interface [40]. As shown in Figure 12c, as the tensile strain increased, the major crack continued to propagate along the direction perpendicular to the tensile direction.

Irwin described the stresses and displacements near the crack-tip by a single constant that was related to the energy release rate, known as the “stress-intensity factor” [52]:

(2) $$K\;=\;Y\sigma \sqrt{\pi a}$$

where *Y* is dimensionless constant that depends on the geometry and the mode of loading, *σ* is the characteristic stress and *a* is the characteristic crack dimension. When *Y* and *σ* are set as constants, the larger is the crack dimension *a*, the larger the stress-intensity factor *K*, representing the power to motivate the propagation of crack. When the factor *K* is beyond a critical value, *K*_IC_, the crack propagation will occur in an unstable manner. This explains why the major crack initiated at the interface between the FG and CG regions underwent an unstable propagation, resulting in the ultimate failure finally (Figure 12d).

## 5. Conclusions

In this paper, the deformation behavior and the fracture mechanism, as well as their relationship of an as-extruded Mg-Gd-Y alloy with bimodal structure was studied. In-situ tensile tests combined with EBSD orientation analysis were used to reveal the slip activities, deformation and fracture behaviors of the alloy during tensile deformation. The following conclusions can be drawn:

(1) The specimen showed good strength-ductility synergy, showing relatively long uniform deformation stage. The fracture surface of the specimen showed the ductile features. The main slip modes in the fine DRXed grains and in the coarse hot-worked grains were basal slips and prismatic slips, respectively.
(2) The increment of the average GND density of the FG region was greater than that of the CG region during tension. This indicates that the FG region carried more plastic strain. This strain incompatibility led to HDI strengthening and strain hardening, conducing to the improvement of strength-ductility combination.
(3) The crack nucleation occurred at the grain boundaries in the FG region, as well as at the interface between the FG and CG regions. The propagation of the microcracks in the FG region was hindered, because of the toughening effect of the fine DRXed grains. The final fracture of the sample was caused by the unstable propagation of the major crack at the interface between the two domains, facilitated by the high stress concentration and the large crack size.

The strength-ductility balance and the associated deformation and fracture behaviors significantly determine the performance of Mg alloys in engineering applications. This work could provide guidance for choosing or designing microstructural features in Mg-RE alloys to meet the demand in service. For this purpose, further studies are required to focus on the fracture toughness and the underlying mechanisms of the bimodal- or hetero-structured Mg-RE alloys.

## Figures and Tables

**Table 1:** Tensile properties of the EX and as-solution specimens at room temperature.

Specimen	Tensile Strength (MPa)	Yield Strength (MPa)	Elongation (%)
EX	288.8 ± 4.8	212.8 ± 1.8	9.8 ± 0.4
as-solution	103.5 ± 3.6	78.5 ± 1.5	1.7 ± 0.3

## Data Availability

Data will be made available on request.

## Abbreviations

EX: as-extruded; DRXed: dynamic recrystallized; GNDs: geometrically necessary dislocations; HDI: hetero-deformation induced; Mg-RE alloys: rare-earth magnesium alloys; EBSD: electron backscatter diffraction; ICP-AES: inductively coupled plasma-atomic emission spectrometer; XRD: X-ray diffraction; PF: pole figure; ED: extrusion direction; SEM: scanning electron microscope; SE: secondary electron; TD: transverse direction; SHR: strain hardening rate; FG: fine-grained; CG: coarse-grained; HAADF: high-angle annular dark field; STEM: scanning transmission electron microscopy; EDS: energy dispersive spectrometer; SF: schmid factor; IPF: inverse pole figure; KAM: kernel average misorientation; CI: confidential index; CRSS: critical resolved shear stress.
